# Genetic engineering of *AtAOX1a* in *Saccharomyces cerevisiae* prevents oxidative damage and maintains redox homeostasis

**DOI:** 10.1002/2211-5463.12028

**Published:** 2016-02-01

**Authors:** Abhaypratap Vishwakarma, Ahan Dalal, Sarada Devi Tetali, Pulugurtha Bharadwaja Kirti, Kollipara Padmasree

**Affiliations:** ^1^ Department of Plant SciencesSchool of Life SciencesUniversity of HyderabadHyderabadIndia; ^2^ Department of Biotechnology and BioinformaticsSchool of Life SciencesUniversity of HyderabadHyderabadIndia

**Keywords:** alternative oxidase 1a, oxidative stress, reactive oxygen species, redox homeostasis, respiration, *Saccharomyces cerevisiae*

## Abstract

This study aimed to validate the physiological importance of *Arabidopsis thaliana* alternative oxidase 1a (AtAOX1a) in alleviating oxidative stress using *Saccharomyces cerevisiae* as a model organism. The AOX1a transformant (pYES2AtAOX1a) showed cyanide resistant and salicylhydroxamic acid (SHAM)‐sensitive respiration, indicating functional expression of *AtAOX1a* in *S. cerevisiae*. After exposure to oxidative stress, pYES2AtAOX1a showed better survival and a decrease in reactive oxygen species (ROS) when compared to *S. cerevisiae* with empty vector (pYES2). Furthermore, pYES2AtAOX1a sustained growth by regulating *GPX2* and/or *TSA2,* and cellular NAD ^+^/NADH ratio. Thus, the expression of *AtAOX1a* in *S. cerevisiae* enhances its respiratory tolerance which, in turn, maintains cellular redox homeostasis and protects from oxidative damage.

**Abbreviations**
ADH: alcohol dehydrogenase
AOX: alternative oxidase
AtAOX1a: *Arabidopsis thaliana* alternative oxidase 1a
COX: cytochrome *c* oxidase
DCPIP: dichlorophenolindophenol
IPTG: isopropyl‐β‐d‐thiogalactopyranoside
PCD: programmed cell death
PG: propyl gallate
PMS: phenazine methosulfate
PVDF: polyvinylidene difluoride
ROS: reactive oxygen species
SHAM: salicylhydroxamic acid
SOD: superoxide dismutase
t‐BOOH: tert‐butyl hydroperoxide
TCA: tricarboxylic acid
UQ: ubiquinone

Alternative oxidase (AOX) is a nonproton pumping ubiquinol oxidase localized in the inner mitochondrial membrane of higher plants, fungi, some protists and was recently identified in 28 animal species 1. In contrast to cytochrome *c* oxidase (COX), it is cyanide resistant and branches from the ‘standard’ mitochondrial respiratory chain at the level of ubiquinone (UQ). It is considered as a sink for excess electrons as it reduces the molecular oxygen to water, bypassing the oxidative phosphorylation at both complex III and IV. Thus, AOX plays an important role in maintaining the cellular energy balance 2, 3, 4. A crystal structure of AOX from *Trypanosome brucei* revealed that it is a homodimer, which exists as an integral interfacial membrane protein with a nonhaem diiron carboxylate active site buried within a four helix bundle. The active site is ligated by four glutamate residues and a highly conserved Tyr220, which mediates its catalytic activity. Furthermore, the two hydrophobic cavities occur per monomer which bind to ubiquinol and Tyr220 for catalytic cycle and O_2_ reduction 5, 6, 7.

AOX was first identified in thermogenic plants to provide favorable temperature during floral development to attract pollinators 8, 9, 10, 11. In nonthermogenic plants, AOX is known to prevent over‐reduction of UQ and generation of reactive oxygen species (ROS) while allowing continued operation of the tricarboxylic acid (TCA) cycle 12, 13, 14. On exposure to abiotic stress, AOX‐deficient plants showed an increase in intracellular ROS and a decrease in photosynthetic performance as compared to wild‐type plants 15, 16, 17, 18, 19. On the other hand, AOX overexpression lines showed an enhanced photosynthetic efficiency with lower levels of cellular ROS when compared with wild‐type plants during abiotic stress conditions 20, 21, 22. In *Arabidopsis,* overexpression of AOX1a alleviated the Al‐induced programmed cell death (PCD) by decreasing the ROS production due to efficient mitochondrial electron flux and caspase‐3‐like activation 23. Also, the role of AOX has been studied extensively in lower organisms since last two decades. Kumar and Söll 24 reported for the first time heterologous expression of *Arabidopsis thaliana* AOX into *hemA*‐deficient strains of *Escherichia coli*, which acquired resistance to cyanide and exhibited aerobic respiration. Later, several other studies also demonstrated the expression of AOX in many yeast, fungal, and bacterial species, which resulted in the successful operation of cyanide‐insensitive respiration 25, 26, 27, 28, 29, 30. Recently, Honda *et al*. 29 demonstrated the visual expression of AOX in *Aspergillus niger* transformants (harboring fusion gene *aox1‐egfp*) upon exposure to heat shock, oxidative, and osmotic stress. Furthermore, expression of AOX from *Hansenula anomala* in *Saccharomyces cerevisiae* resulted in up‐regulation of several proteins related to major metabolic pathways such as Krebs cycle and amino acid biosynthesis suggesting the physiological role of AOX in mitoproteome plasticity 31. The role of AOX is also revealed in the survival of pathogenic fungi such as *Aspergillus fumigatus* and *Histoplasma capsulatum* inside the host under stress conditions 32, 33. Similar to plants, the AOX mutant of pathogenic yeast *Cryptococcus neoformans* showed susceptibility to oxidative stress 34.

Yeast cells have become one of the most preferred experimental models to study the PCD and aging under oxidative stress, owing to special characteristics such as short life cycle and ease for genetic manipulation along with presence of core cellular processes similar to eukaryotes 35. In most of the aerobic cells, respiration is the major source for generation of superoxide radical (O_2_^−^) as electrons leak out from the mitochondrial electron transport chain at Complex I and Complex III. Furthermore, dismutation of O_2_^−^ by superoxide dismutase (SOD) generates H_2_O_2_, a quite stable toxic product which creates oxidative environment inside the cell 36. To detoxify the cellular H_2_O_2_, mitochondria have evolved an efficient antioxidant defense system such as catalase and peroxiredoxins, which include glutathione peroxidase/glutathione reductase and thioredoxin peroxidase/thioredoxin reductase 37. In spite of the existence of such a strong antioxidant defense system, several *pet* mutants (impaired in mitochondrial electron transport chain) of *S. cerevisiae* showed accumulation of H_2_O_2_. However, the addition of exogenous cytochrome c to isolated mitoplasts significantly decreased the H_2_O_2_ levels 38. In *Candida albicans* and *Aspergillus niger*, AOX was also induced along with cytochrome c under oxidizing conditions 30, 39. Thus, AOX pathway is known to play an important role in the alleviation of ROS and thereby oxidative stress, either independently or in association with the COX pathway and/or antioxidant defense system. Furthermore, a direct or an indirect role of AOX has also been demonstrated in maintaining redox homeostasis in higher plants in response to several abiotic stresses 18, 19, 40, 41, 42. However, such type of significance for AOX is yet to be elucidated in lower organisms.

In *Arabidopsis,* AOX1a is known to be induced under various oxidative stresses (imposed by biotic and abiotic stresses) and developmental stages 15, 16, 18, 43, 44, 45, which indicate that genetic engineering of *AOX1a* might be a promising tool to combat oxidative stress in AOX deficient strains or organisms. In the present study, *AtAOX1a* was heterologously expressed in *S. cerevisiae* (an eukaryotic organism devoid of AOX) to characterize its role in response to oxidative stress. To create an oxidative environment inside the cells, *S. cerevisiae* were incubated with H_2_O_2_ and tertiary‐butyl hydroperoxide (t‐BOOH). The functional expression of *AtAOX1a* and its characterization have been studied by monitoring the changes in respiration, growth, viability, ROS, antioxidant system, and redox state of *S. cerevisiae* under oxidizing conditions.

## Materials and methods

### Strains and culture conditions

*Escherichia coli* (*E. coli*) DH5α or BL21(DE3)pLysS (Invitrogen^™^, Waltham, MA, USA) were grown at 37 °C in Luria–Bertani medium. *Saccharomyces cerevisiae* strain INVSc1 (Invitrogen^™^) was grown at 30 °C either in YPD medium (1% w/v yeast extract, 2% w/v peptone and 2% w/v dextrose) or SC‐URA¯ minimal medium (0.67% w/v yeast nitrogen base without amino acids, 2% w/v glucose as carbon source) and amino acids.

### Cloning of AtAOX1a and plasmid construction

Total RNA was isolated from *A. thaliana* wild‐type leaves using TRI reagent (Sigma‐Aldrich, St. Louis, MO, USA). One microgram of total RNA was used for the first‐strand cDNA synthesis using iScript^™^ cDNA synthesis kit (Bio‐Rad, Hercules, CA, USA). *AtAOX1a* encoding a mature protein was amplified by Phusion DNA polymerase (Clontech, CA, USA) using the following primers: F‐GAGAATTCGCTAGCACGATCACTCTGG and R‐GGCTCGAGTCAATGATACCCAATTGGAG, and cloned into a pET28a(+)^™^ expression vector. In contrast, *AtAOX1a* encoding a mature protein along with its leader sequence was amplified by using the primers: F‐GGGAATTCTGATGATGATAACTCGCGGTGG and R‐GGCTCGAGTCAATGATACCCAATTGGAG, and cloned into a pYES2/NT expression vector. Clones were confirmed by DNA sequencing. The recombinant plasmids were transformed into their respective host strains, i.e., BL21(DE3)pLysS and INVSc1.

### Protein expression, purification, and antibody generation

The expression of AtAOX1a in *E. coli* BL21(DE3)pLysS was induced by 0.1 mm isopropyl‐β‐d‐thiogalactopyranoside (IPTG) at 28 °C for 4 h. The recombinant protein was purified under denaturing conditions with Ni‐NTA agarose column using standard protocols and the purified protein from the gel slice was subjected to a matrix‐assisted laser desorption ionization time‐of‐flight mass spectrometry (MALDI‐TOF/TOF) analysis as described in ref. 46 for confirmation as AtAOX1a. The purified protein was used to generate a polyclonal antibody in rabbit using standard protocols (Animal ethics approval number is UH/IAEC/KPMS/2014‐1/24).

### AtAOX1a protein expression in *Saccharomyces cerevisiae*

For heterologous protein expression, *S. cerevisiae* with empty vector (pYES2) or transformed with *AtAOX1a* (pYES2AtAOX1a) were grown overnight in SC‐URA¯ minimal media containing 2% galactose as a carbon source. Protein was extracted using trichloroacetic acid (TCA) method 47 and separated on a 12.5% SDS/PAGE. For immunodetection, protein gel was electroblotted onto polyvinylidene difluoride (PVDF) membrane and treated with a polyclonal AOX1a antibody (generated as mentioned in section ‘Protein expression, purification, and antibody generation’) at 1 : 1000 dilutions followed by a goat anti‐rabbit IgG‐alkaline phosphate conjugate (Sigma, USA) at 1 : 5000 dilutions. The blot was developed using 5‐bromo‐4‐chloro‐3‐indolyl phosphate/nitro blue tetrazolium (BCIP/NBT) system.

### Oxidative stress

Oxidative stress analyses were performed as described earlier 48. Treatment duration was different for each set of experiments depending on their feasibility. The duration of oxidative stress treatment was fixed at 10 min for ROS estimation, 4 h for survival rate and growth recovery assay, and 75 min for pyridine nucleotides and transcript level analyses.

### Measurement of O_2_ uptake and cell survival rate

The respiratory O_2_ uptake measurements (10 min) were performed using Clark‐type O_2_ electrode 49, 50. The viability of cells was examined with fluctuation assay as reported by Dalal *et al*. 50.

### Measurement of ROS

The intracellular ROS level was measured following Jang *et al*. 51. The cells were incubated with 100 μm 2′,7′‐ dichlorodihydrofluorescein diacetate (H_2_DCF‐DA; Sigma) for 5 min in the dark at 25 °C, and the change in DCF fluorescence was imaged under a laser‐scanning confocal fluorescence microscope (LSM 710 NLO ConfoCor 3; Carl Zeiss, Jena, Germany).

### Measurement of pyridine nucleotide content

The extraction and estimation of NAD^+^ and NADH were done as per Queval and Noctor 52. The assay involves phenazine methosulfate (PMS) catalyzed reduction of dichlorophenolindophenol (DCPIP) in the presence of alcohol dehydrogenase (ADH) and ethanol. The NAD^+^ and NADH content were calculated using the relevant standard (0–40 pmole).

### RNA isolation and expression analysis

Total RNA was isolated using the acid‐phenol method 53. First strand cDNA was synthesized with 2 μg of total RNA using SuperScript^®^ III (Invitrogen) according to manufacturer's instructions. Primers used for real‐time PCR analysis are listed in Table 1 42. Comparative *C*_T_ method was used to analyze the relative gene expression levels 54.

**Table 1:** List of primers used in real‐time PCR study. *ACT1* was used as housekeeping gene

Gene	Accession no.	Primer sequence (5′ to 3′)	Amplicon length (bp)
*SOD1*	YJR104C	R‐TAACGACGCTTCTGCCTACA	191
*SOD2*	YHR008C		191
*GPX2*	YBR244W		172
*TSA2*	YDR453C		159
*ACT1*	YFL039C		184

### Statistical analysis

All values are presented as means ± standard errors of the means (SEM). The statistical evaluation of the data was performed with one‐way analysis of variance (ANOVA), Tukey test of multiple comparison analysis using sigma plot 11.0 software (Systat, San Jose, CA, USA). *P* values of < 0.05 were considered as statistically significant.

## Results

### Expression of AtAOX1a in *Escherichia coli* and mass analysis

**Figure 1:**
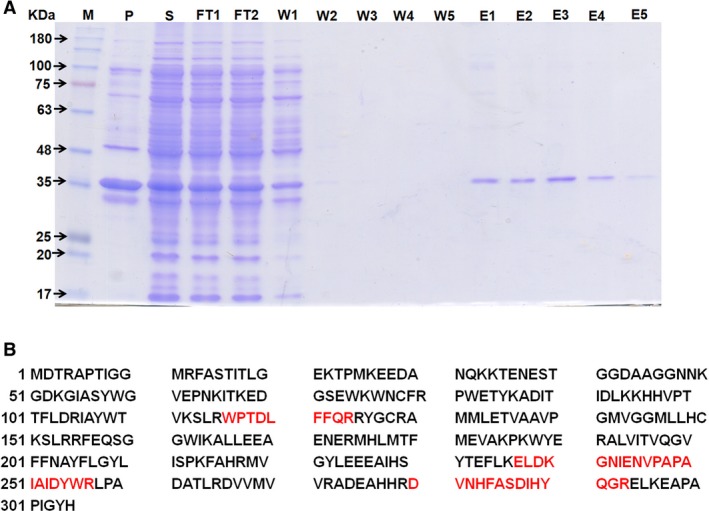
Purification profile and molecular mass analysis of a polyhistidine tagged pET28a‐AtAOX1a recombinant protein: (A) 12.5% SDS/PAGE depicting a ~ 36 kDa AtAOX1a protein in different fractions of the purification protocol. Abbreviations used are as follows: M‐marker, P‐pellet (insoluble protein), S‐supernatant (soluble protein), FT‐flow through (supernatant passed through Ni‐NTA column), W‐washing fractions, E‐elute (purified protein). (B) The sequences corresponding to peptide fragments with molecular masses of 1209.819, 1659.057, 1899.286, and 2384.592 Da, respectively, obtained from MALDI‐TOF‐TOF analysis of trypsin digested purified protein showed 100% matching to internal sequences (indicated in red font) of *Arabidopsis thaliana* AOX1a (AT3G22370), retrieved from NCBI database.

The expression of AtAOX1a protein induced in the presence of 0.1 mm IPTG in *E. coli* was visualized on SDS/PAGE as a ~ 36 kDa band as it includes AtAOX1a sequence encoding a mature protein (32.34 kDa) and pET28a(+) vector sequence (3.83 kDa) (Fig. 1A). Four major peptide fragments obtained during MALDI‐TOF‐TOF analysis of a trypsin‐digested protein showed the following sequences in Biotools: WPTDLFFQR (1209.81 Da), DVNHFASDIHYQGR (1658.04 Da), GNIENVPAPAIAIDYWR (1898.27 Da), and ELDKGNIENVPAPAIAIDYWR (2383.58 Da). As the sequences from these peptides showed 100% matching with *Arabidopsis* AOX1a (Fig. 1B, and Figs S1, S2A–D), the purified protein was injected into a rabbit and the polyclonal antibody was obtained.

### Functional characterization of AtAOX1a in *Saccharomyces cerevisiae*

**Figure 2:**
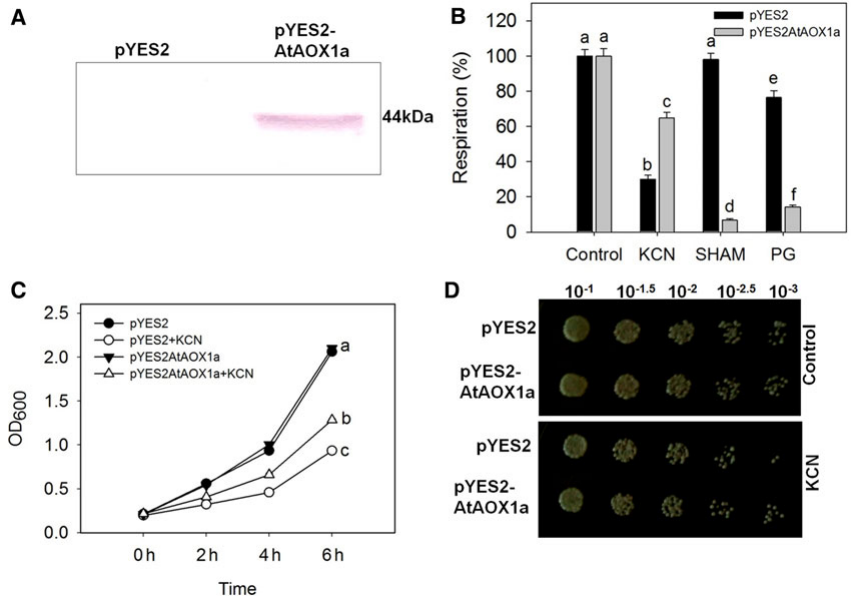
Functional expression of AtAOX1a in *Saccharomyces cerevisiae*. (A) Western blot showing the AtAOX1a protein (44 kDa) expression in pYES2AtAOX1a (right side) but not in PYES2 (left side); (B) Rates of oxygen uptake by pYES2 and pYES2AtAOX1a in the absence or presence of KCN (1 mm), SHAM (2 mm), and PG (100 μm); (C) Time‐dependent growth curve of pYES2 and pYES2AtAOX1a in the absence or presence of KCN (1 mm) and (D) Growth recovery in pYES2 and pYES2AtAOX1a after KCN (1 mm) treatment for 4 h. Different lowercase alphabetical letters indicate statistically significant difference (*P* < 0.05).

The protein expression of AtAOX1a in *S. cerevisiae* was confirmed through western blot analysis (Fig. 2A). To ascertain the function of AtAOX1a, cyanide‐sensitive respiration was monitored using 1 mm KCN, an inhibitor of complex IV in COX pathway, while cyanide‐insensitive respiration was monitored in the presence of 2 mm salicylhydroxamic acid (SHAM) or 100 μm propyl gallate (PG), inhibitors of AOX in the alternative pathway. In the absence of metabolic inhibitors, the respiratory rates of pYES2AtAOX1a (8.6 ± 0.11 nmol O_2_ s^−1^) were similar to pYES2 (8.45 ± 0.09 nmol O_2_ s^−1^). But, in the presence of KCN, pYES2 showed a pronounced decrease in respiratory rates when compared with pYES2AtAOX1a. In contrast, addition of SHAM or PG significantly decreased the respiratory rates of pYES2AtAOX1a but not of pYES2 (Fig. 2B).

The exponential growth pattern of both pYES2 and pYES2AtAOX1a were found to be similar (OD_600_ = 2.1) up to 6 h. But, treatment with KCN remarkably decreased the exponential growth in yeast cells (Fig. 2C). However, the decrease in exponential growth of pYES2 was more significant when compared with pYES2AtAOX1a. Furthermore, in the presence of KCN, growth recovery was found to be higher in pYES2AtAOX1a than pYES2 (Fig. 2D). Taken together, these results indicate that AtAOX1a was successfully expressed and functional in *S. cerevisiae*.

### Changes in cellular ROS during oxidative stress

**Figure 3:**
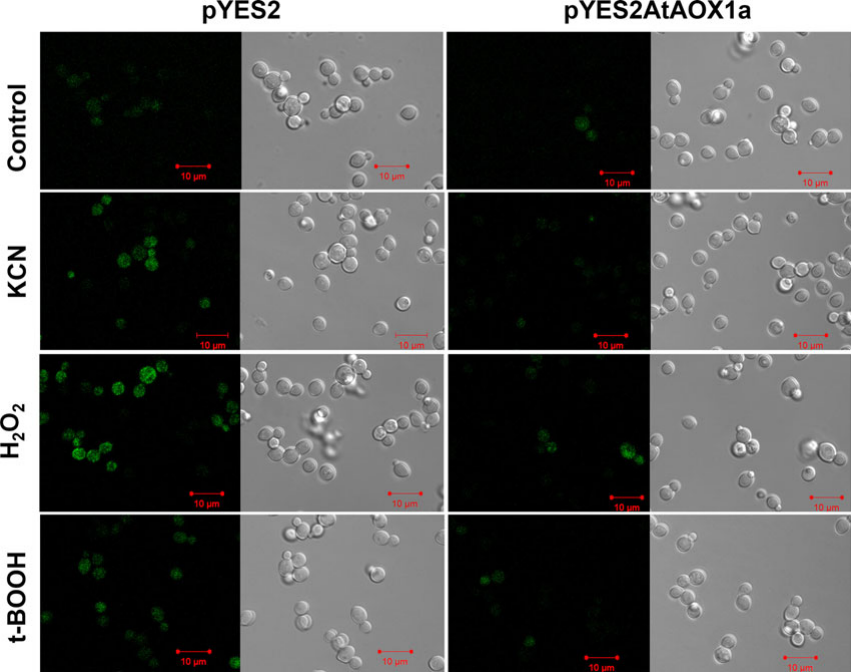
Effect of H_2_O_2_ (2 mm) or t‐BOOH (0.25 mm) on the intracellular ROS generation. ROS were monitored in pYES2 and pYES2AtAOX1a at 488 nm (excitation) and 525 nm (emission) wavelengths under a confocal fluorescence microscope as DCF fluorescence produced by the action of esterases on H_2_ DCFDA. Sample treated with KCN (1 mm) was used as a positive control.

Under control conditions, the cellular ROS was minimal in both pYES2 and pYES2AtAOX1a as indicated by DCF fluorescence. However, upon treatment with KCN, H_2_O_2_, or t‐BOOH, the fluorescence increased significantly in pYES2. In contrast, pYES2AtAOX1a restricted the increase in fluorescence during oxidative stress indicating the importance of AOX1a in preventing and/or regulating the ROS generation (Fig. 3).

### Changes in cell survival rate and growth recovery during oxidative stress

**Figure 4:**
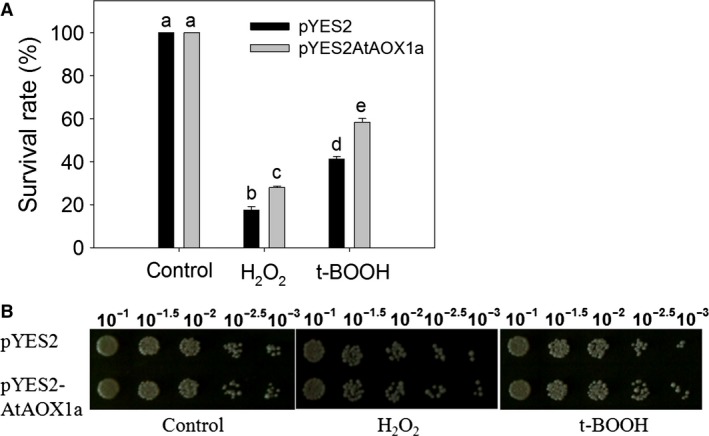
Effect of H_2_O_2_ (2 mm) or t‐BOOH (0.25 mm) on (A) the cell survival rate and (B) return to growth assay in pYES2 and pYES2AtAOX1a. Different lowercase alphabetical letters indicate statistically significant difference (*P* < 0.05).

Among the two oxidants, H_2_O_2_ was found to be more lethal than t‐BOOH. Upon treatment with these oxidants, the survival rate of pYES2 decreased drastically as compared to pYES2AtAOX1a (Fig. 4A). Also, recovery assays clearly indicated an enhanced colony number in pYES2AtAOX1a than in pYES2 under oxidizing conditions with a clear visible difference at 1 × 10^−2.5^ and 1 × 10^−3^ dilutions (Fig. 4B). It appears that AOX1a plays a critical role in decreasing the rates of cell death and improving their growth recovery under oxidizing conditions.

### Differential antioxidant gene expression profile during oxidative stress

**Figure 5:**
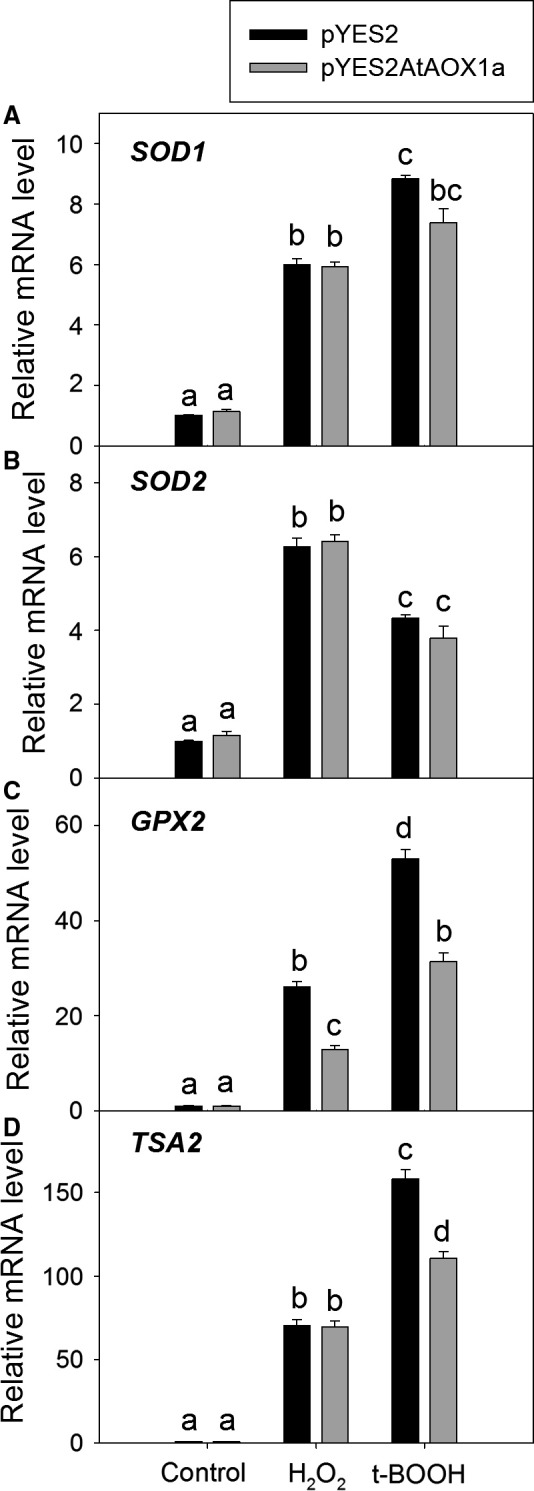
Relative mRNA profile of the antioxidant genes (A) *SOD1*, (B) *SOD2*, (C) *GPX2*, and (D) *TSA2* in pYES2 and pYES2AtAOX1a after exposure to H_2_O_2_ (2 mm) or t‐BOOH (0.25 mm). *ACT1* was used as housekeeping gene. Different lowercase alphabetical letters indicate statistically significant difference (*P* < 0.05).

The ROS scavenging efficiency of pYES2 and pYES2AtAOX1a was measured by monitoring the changes in transcript levels of antioxidant genes viz., *Superoxide dismutase 1* (*SOD1*), *Superoxide dismutase 2* (*SOD2*), *Glutathione peroxidase 2* (*GPX2*), and *Thioredoxin peroxidase 2* (*TSA2*) during oxidative stress (Fig. 5A–D). Under control conditions, the expression of these antioxidant genes was approximately similar in both pYES2 and pYES2AtAOX1a. Upon treatment with H_2_O_2_ or t‐BOOH, the expression of *SOD1* (> 8‐fold), *SOD2* (> 6‐fold)*, GPX2* (> 52‐fold), and *TSA2* (> 157‐fold) increased significantly by several fold in both pYES2 and pYES2AtAOX1a (Fig. 5A–D). But, the expression of *GPX2* was down‐regulated significantly in pYES2AtAOX1a when compared with pYES2 in the presence of both H_2_O_2_ and t‐BOOH (Fig. 5C). In contrast, the expression of *TSA2* was down‐regulated significantly in pYES2AtAOX1a when treated with t‐BOOH, while remained unchanged in the presence of H_2_O_2_ (Fig. 5D).

### Changes in cellular redox during oxidative stress

**Figure 6:**
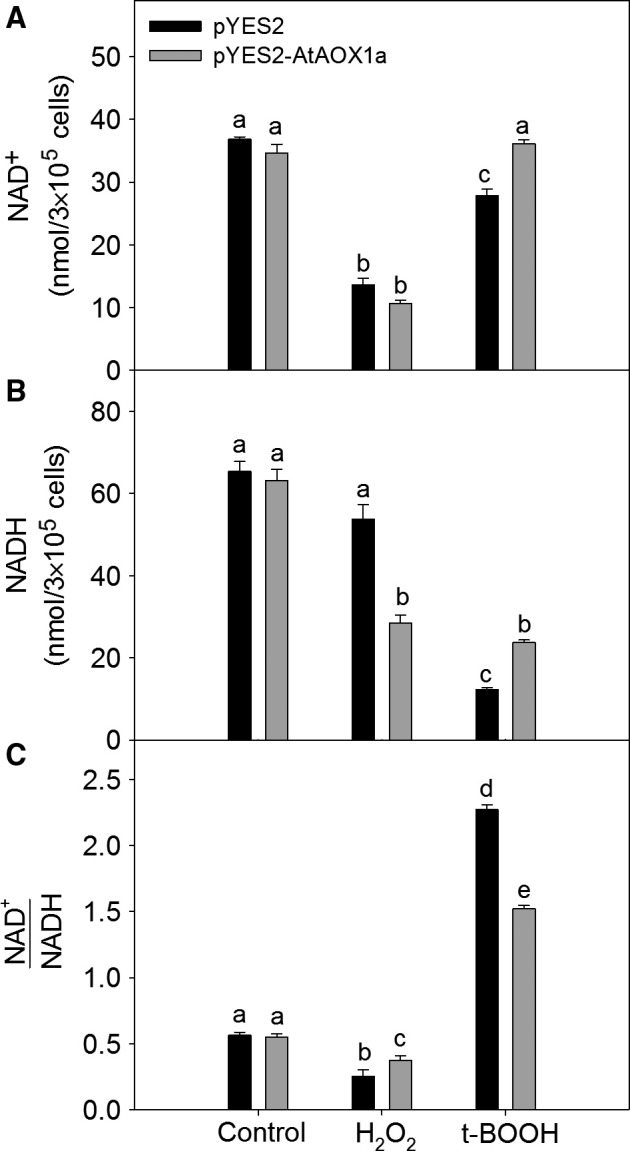
Changes in the total cellular pyridine nucleotides (A) NAD ^+^; (B) NADH; and (C) ratio of NAD ^+^ to NADH in pYES2 and pYES2AtAOX1a upon treatment with H_2_O_2_ (2 mm) or t‐BOOH (0.25 mm). Different lowercase alphabetical letters indicate statistically significant difference (*P* < 0.05).

The role of AtAOX1a in maintaining the cellular redox balance during oxidative stress was revealed by monitoring the changes in pyridine nucleotide (NAD^+^ and NADH) redox couple. In control, the cellular levels of NAD^+^, NADH, and the redox ratio of NAD^+^/NADH were similar in both pYES2 and pYES2AtAOX1a. Upon treatment with H_2_O_2_, the cellular NAD^+^ levels decreased significantly in both pYES2 and pYES2AtAOX1a (Fig. 6A). In contrast, the decrease in cellular NADH levels was significant in pYES2AtAOX1a alone (Fig. 6B). Consequently, the cellular redox ratio of NAD^+^/NADH was maintained at much higher levels in pYES2AtAOX1a when compared with pYES2 in the presence of H_2_O_2_ (Fig. 6C).

The responses of NAD^+^, NADH, and consequently NAD^+^/NADH were quite different in t‐BOOH‐treated samples as compared to H_2_O_2_ treatment. In the presence of t‐BOOH, both NAD^+^ and NADH levels increased significantly, while the redox ratio of NAD^+^/NADH decreased drastically in pYES2AtAOX1a when compared with pYES2 (Fig. 6A–C).

## Discussion

In higher plants, AOX is known to perform several mitochondrial and extramitochondrial functions, viz: (a) alleviation of reactive oxygen and nitrogen species, and cell death 16, 55, 56, 57, (b) preventing over‐reduction of chloroplastic/mitochondrial electron transport carriers, particularly plastoquinone or UQ 13, (c) maintenance of cellular redox and carbon balance 18, 19, 58, (d) modulation of cellular energy level 59, and (e) optimization of photosynthesis during a wide range of biotic and abiotic stresses 18, 19, 60, 61. The role of AOX in alleviating ROS levels and oxidative stress is not only confined to plants but was also revealed in several nonphotosynthetic organisms including fungi, protists, bacteria, and human cells 29, 39, 62, 63. These observations suggest that engineering of AOX into such species which are deficient in AOX may help them to cope up against various biotic and abiotic stresses.

*Saccharomyces cerevisiae* lacks an AOX homolog 64. Therefore, *AtAOX1a* was expressed in *S. cerevisiae* to validate its physiological function during oxidative stress (Fig. 2A). It is well known that any restriction of electron flow through the COX pathway or exposure to oxidative stress leads to an induction of AOX in plants and fungi 21, 27, 60, 65. Corroborating with these studies, restriction of electron transport through the COX pathway by KCN caused a significant reduction in the total respiratory rates of pYES2 and pYES2AtAOX1a. However, due to an AOX catalyzed respiration, pYES2AtAOX1a showed higher respiratory rates compared to pYES2. While the SHAM‐insensitive respiration in pYES2 indicates the absence of AOX‐catalyzed respiration, SHAM or PG‐sensitive respiration in pYES2AtAOX1a confirms the functional expression of AtAOX1a in yeast (Fig. 2B) 29, 39. Any increase in the respiratory activity is known to increase the chronological and replicative lifespan of yeast 66. Also, the recovery in the growth curve assays and a rise in the total respiratory rates of pYES2AtAOX1a in the presence of KCN reveal the significance of AOX‐catalyzed respiration in the maintenance of yeast cell growth (Fig. 2B–D).

ROS production is a common phenomenon in cells, which occurs during aerobic respiration or in response to several biotic or abiotic stresses. But, excessive ROS production leads to oxidative stress 67, 68, 69. Yeast cells show a range of responses depending on the concentration of cellular ROS. At very low levels of ROS, the cells try to adapt themselves, while at higher levels of ROS, the cells activate their antioxidant defense system mediated by Yap1p and Msn2,4p transcription factors 70. Beyond this, ROS might arrest the cell cycle leading to apoptosis 71, 72. In the present study, the higher levels of cellular ROS induced by KCN, H_2_O_2_, or t‐BOOH in pYES2 were positively correlated with cell death and negatively correlated with growth recovery. In contrast, the lower levels of ROS, better survival rate, and growth recovery recorded under oxidizing environment in pYES2AtAOX1a indicate the importance of AOX catalyzed respiration in mitigating the cellular ROS production (Figs 3 and 4A,B).

Redox homeostasis is a basic requirement to maintain the cellular metabolism and ROS, particularly during aging 72, 73. Accumulation of NADH decreases the Sir2 activity, which is essential for chromatin silencing and extension of life span. Thus, any increase in the redox ratio of NAD^+^/NADH extended the chronological as well as replicating life span of yeast cells 74, 75. The pYES2AtAOX1a showed an increase in the NAD^+^/NADH ratio when compared with pYES2 upon treatment with H_2_O_2_. In contrast, pYES2AtAOX1a maintained the cellular redox homeostasis by minimizing the redox ratio of NAD^+^/NADH raised by t‐BOOH. These results elucidate the importance of AtAOX1a in the maintenance of cellular redox homeostasis to increase the life span as evident by cell survival rate of yeast (Figs 4A,B and 6C).

Furthermore, the sulphydryl (‒SH) group plays a critical role in proper functioning of several of the enzymes, transcription factors, and membrane proteins, which in turn play a significant role in maintaining the cellular redox homeostasis 73. During oxidative stress, cysteine sulfhydryl residues are oxidized to disulfide bonds, thereby leading to a loss in protein activity. Small heat‐stable oxidoreductases, glutaredoxins, and thioredoxins catalyze the reduction of disulfides to thiols using thiolated cysteine residues present in the active sites 73, 76, 77. A few studies reported the role of glutaredoxins and thioredoxins in supplying reducing equivalents to the regulatory sulfhydryl/disulfide system of AOX to activate it, which in turn play a role in preventing the over‐reduction of mitochondrial electron transport carriers and thereby ROS generation 78, 79, 80. In the present study, a several fold increase in the transcript levels of *GPX2* and *TSA2* in pYES2 and their down‐regulation in pYES2AtAOX1a in the presence of t‐BOOH and/or H_2_O_2_ suggests the role of AOX1a in regulating the expression of these antioxidant enzymes, which play an important role in the detoxification of ROS and the maintenance of cellular redox balance (Figs 3, 5C,D and 6C).

The results from the present study suggest that transformation of *AtAOX1a* introduced AOX‐catalyzed respiration in *S. cerevisiae*, which in turn mitigated ROS generation by regulating *GPX2* and *TSA2* to maintain cellular redox homeostasis and better cell survival rate during oxidative stress.

## Author contributions

KP conceived and supervised the study; KP, SDT and AV designed the experiments; AV and AD acquired the data; KP, SDT, PBK and AV analyzed and interpreted the data; AV wrote the paper; KP edited the paper; SDT and PBK contributed important intellectual content.

## Supporting information


**Fig. S1** MALDI‐TOF‐TOF mass spectrum of trypsin digested purified AtAOX1a protein between 500 and 5000 m/z.
**Fig. S2** Lift spectrum and Biotools display of four major peaks from trypsin digested AtAOX1a protein: (A) m/z 1209.819, (B) 1659.057, (C) 1899.286, and (D) 2384.592.

## References

[1] McDonald AE , Vanlerberghe GC and Staples JF (2009) Alternative oxidase in animals: unique characteristics and taxonomic distribution. J Exp Biol 212, 2627–2634. 19648408 10.1242/jeb.032151

[2] Moore AL and Siedow JN (1991) The regulation and nature of the cyanide‐resistant alternative oxidase of plant mitochondria. Biochim Biophys Acta 1059, 121–140. 1883834 10.1016/s0005-2728(05)80197-5

[3] Vanlerberghe GC and McIntosh L (1997) Alternative oxidase: from gene to function. Annu Rev Plant Biol 48, 703–734. 10.1146/annurev.arplant.48.1.703 15012279

[4] Millenaar F and Lambers H (2003) The alternative oxidase: *in vivo* regulation and function. Plant Biol 5, 2–15.

[5] Moore AL , Shiba T , Young L , Harada S , Kita K and Ito K (2013) Unraveling the heater: new insights into the structure of the alternative oxidase. Annu Rev Plant Biol 64, 637–663. 23638828 10.1146/annurev-arplant-042811-105432

[6] Shiba T , Kido Y , Sakamoto K , Inaoka DK , Tsuge C , Tatsumi R , Takahashi G , Balogun EO , Nara T , Aoki T *et al* (2013) Structure of the trypanosome cyanide‐insensitive alternative oxidase. Proc Natl Acad Sci USA 110, 4580–4585. 23487766 10.1073/pnas.1218386110 PMC3607012

[7] Young L , Shiba T , Harada S , Kita K , Albury MS and Moore AL (2013) The alternative oxidases: simple oxidoreductase proteins with complex functions. Biochem Soc Trans 41, 1305–1311. 24059524 10.1042/BST20130073

[8] Meeuse BJ (1975) Thermogenic respiration in aroids. Annu Rev Plant Physiol 26, 117–126.

[9] Watling JR , Robinson SA and Seymour RS (2006) Contribution of the alternative pathway to respiration during thermogenesis in flowers of the sacred lotus. Plant Physiol 140, 1367–1373. 16461386 10.1104/pp.105.075523 PMC1435819

[10] Wagner AM , Krab K , Wagner MJ and Moore AL (2008) Regulation of thermogenesis in flowering Araceae: the role of the alternative oxidase. Biochim Biophys Acta 1777, 993–1000. 18440298 10.1016/j.bbabio.2008.04.001

[11] Miller RE , Grant NM , Giles L , Ribas‐Carbo M , Berry JA , Watling JR and Robinson SA (2011) In the heat of the night–alternative pathway respiration drives thermogenesis in *Philodendron bipinnatifidum* . New Phytol 189, 1013–1026. 21118259 10.1111/j.1469-8137.2010.03547.x

[12] Maxwell DP , Wang Y and McIntosh L (1999) The alternative oxidase lowers mitochondrial reactive oxygen production in plant cells. Proc Natl Acad Sci USA 96, 8271–8276. 10393984 10.1073/pnas.96.14.8271 PMC22224

[13] Yoshida K , Watanabe CK , Hachiya T , Tholen D , Shibata M , Terashima I and Noguchi K (2011) Distinct responses of the mitochondrial respiratory chain to long‐ and short‐term high‐light environments in *Arabidopsis thaliana* . Plant, Cell Environ 34, 618–628. 21251020 10.1111/j.1365-3040.2010.02267.x

[14] Araújo WL , Nunes‐Nesi A and Fernie AR (2014) On the role of plant mitochondrial metabolism and its impact on photosynthesis in both optimal and sub‐optimal growth conditions. Photosynth Res 119, 141–156. 23456269 10.1007/s11120-013-9807-4

[15] Giraud E , Ho LH , Clifton R , Carroll A , Estavillo G , Tan YF , Howell KA , Ivanova A , Pogson BJ , Millar AH *et al* (2008) The absence of ALTERNATIVE OXIDASE1a in *Arabidopsis* results in acute sensitivity to combined light and drought stress. Plant Physiol 147, 595–610. 18424626 10.1104/pp.107.115121 PMC2409015

[16] Strodtkötter I , Padmasree K , Dinakar C , Speth B , Niazi PS , Wojtera J , Voss I , Do PT , Nunes‐Nesi A , Fernie AR *et al* (2009) Induction of the AOX1D isoform of alternative oxidase in *A. thaliana* T‐DNA insertion lines lacking isoform AOX1A is insufficient to optimize photosynthesis when treated with antimycin A. Mol Plant 2, 284–297. 19825614 10.1093/mp/ssn089

[17] Watanabe CK , Hachiya T , Takahara K , Kawai M , Uchimiya H , Uesono Y , Terashima I and Noguchi K (2010) Effects of AOX1a deficiency on plant growth, gene expression of respiratory components, and metabolic profile under low‐nitrogen stress in *Arabidopsis thaliana* plants. Plant Cell Physiol 51, 810–822. 20304787 10.1093/pcp/pcq033

[18] Vishwakarma A , Bashyam L , Senthilkumaran B , Scheibe R and Padmasree K (2014) Physiological role of AOX1a in photosynthesis and maintenance of cellular redox homeostasis under high light in *Arabidopsis thaliana* . Plant Physiol Biochem 81, 44–53. 24560882 10.1016/j.plaphy.2014.01.019

[19] Vishwakarma A , Tetali SD , Selinski J , Scheibe R and Padmasree K (2015) Importance of alternative oxidase pathway in regulating cellular redox and ROS homeostasis to optimize photosynthesis during restriction of cytochrome oxidase pathway in *Arabidopsis thaliana* . Ann Bot 116, 555–569. 26292995 10.1093/aob/mcv122 PMC4578005

[20] Umbach AL , Fiorani F and Siedow JN (2005) Characterization of transformed *Arabidopsis* with altered alternative oxidase levels and analysis of effects on reactive oxygen species in tissue. Plant Physiol 139, 1806–1820. 16299171 10.1104/pp.105.070763 PMC1310561

[21] Abu‐Romman S , Shatnawi M , Hasan M , Qrunfleh I , Omar S and Salem N (2012) cDNA cloning and expression analysis of a putative alternative oxidase *HsAOX1* from wild barley (*Hordeum spontaneum*). Gene Genomic 34, 59–66.

[22] Mhadhbi H , Fotopoulos V , Mylona PV , Jebara M , Aouani ME and Polidoros AN (2013) Alternative oxidase 1 (Aox1) gene expression in roots of *Medicago truncatula* is a genotype‐specific component of salt stress tolerance. J Plant Physiol 170, 111–114. 23079242 10.1016/j.jplph.2012.08.017

[23] Liu J , Li Z , Wang Y and Xing D (2014) Overexpression of ALTERNATIVE OXIDASE1a alleviates mitochondria‐dependent programmed cell death induced by aluminium phytotoxicity in *Arabidopsis* . J Exp Bot 65, 4465–4478. 24863436 10.1093/jxb/eru222

[24] Kumar AM and Söll D (1992) *Arabidopsis* alternative oxidase sustains *Escherichia coli* respiration. Proc Natl Acad Sci USA 89, 10842–10846. 1438286 10.1073/pnas.89.22.10842 PMC50438

[25] Albury MS , Dudley P , Watts FZ and Moore AL (1996) Targeting the plant alternative oxidase protein to *Schizosaccharomyces pombe* mitochondria confers cyanide‐insensitive respiration. J Biol Chem 271, 17062–17066. 8663588 10.1074/jbc.271.29.17062

[26] Affourtit C , Albury MS , Krab K and Moore AL (1999) Functional expression of the plant alternative oxidase affects growth of the yeast *Schizosaccharomyces pombe* . J Biol Chem 274, 6212–6218. 10037707 10.1074/jbc.274.10.6212

[27] Magnani T , Soriani FM , Martins VP , Nascimento AM , Tudella VG , Curti C and Uyemura SA (2007) Cloning and functional expression of the mitochondrial alternative oxidase of *Aspergillus fumigatus* and its induction by oxidative stress. FEMS Microbiol Lett 271, 230–238. 17425662 10.1111/j.1574-6968.2007.00716.x

[28] Martins VP , Dinamarco TM , Soriani FM , Tudella VG , Oliveira SC , Goldman GH , Curti C and Uyemura SA (2011) Involvement of an alternative oxidase in oxidative stress and mycelium‐to‐yeast differentiation in *Paracoccidioides brasiliensis* . Eukaryot Cell 10, 237–248. 21183691 10.1128/EC.00194-10 PMC3067407

[29] Honda Y , Hattori T and Kirimura K (2012) Visual expression analysis of the responses of the alternative oxidase gene (aox1) to heat shock, oxidative, and osmotic stresses in conidia of citric acid‐producing *Aspergillus niger* . J Biosci Bioeng 113, 338–342. 22138384 10.1016/j.jbiosc.2011.10.026

[30] Papagianni M and Avramidis N (2012) Cloning and functional expression of the mitochondrial alternative oxidase gene (aox1) of *Aspergillus niger* in *Lactococcus lactis* and its induction by oxidizing conditions. Enzyme Microb Technol 50, 17–21. 22133435 10.1016/j.enzmictec.2011.09.013

[31] Mathy G , Navet R , Gerkens P , Leprince P , De Pauw E , Sluse‐Goffart CM , Sluse FE and Douette P (2006) *Saccharomyces cerevisiae* mitoproteome plasticity in response to recombinant alternative ubiquinol oxidase. J Proteome Res 5, 339–348. 16457600 10.1021/pr050346e

[32] Tudella VG , Curti C , Soriani FM , Santos AC and Uyemura SA (2004) In situ evidence of an alternative oxidase and an uncoupling protein in the respiratory chain of *Aspergillus fumigatus* . Int J Biochem Cell Biol 36, 162–172. 14592541 10.1016/s1357-2725(03)00194-8

[33] Johnson CH , Prigge JT , Warren AD and McEwen JE (2003) Characterization of an alternative oxidase activity of *Histoplasma capsulatum* . Yeast 20, 381–388. 12673621 10.1002/yea.968

[34] Akhter S , McDade HC , Gorlach JM , Heinrich G , Cox GM and Perfect JR (2003) Role of alternative oxidase gene in pathogenesis of *Cryptococcus neoformans* . Infect Immun 71, 5794–5802. 14500501 10.1128/IAI.71.10.5794-5802.2003 PMC201089

[35] Carmona‐Gutierrez D , Eisenberg T , Buttner S , Meisinger C , Kroemer G and Madeo F (2010) Apoptosis in yeast: triggers, pathways, subroutines. Cell Death Differ 17, 763–773. 20075938 10.1038/cdd.2009.219

[36] Turrens JF (1997) Superoxide production by the mitochondrial respiratory chain. Biosci Rep 17, 3–8. 9171915 10.1023/a:1027374931887

[37] Kowaltowski AJ , de Souza‐Pinto NC , Castilho RF and Vercesi AE (2009) Mitochondria and reactive oxygen species. Free Radic Biol Med 47, 333–343. 19427899 10.1016/j.freeradbiomed.2009.05.004

[38] Barros MH , Netto LE and Kowaltowski AJ (2003) H_2_O_2_ generation in *Saccharomyces cerevisiae* respiratory pet mutants: effect of cytochrome c. Free Radical Biol Med 35, 179–188. 12853074 10.1016/s0891-5849(03)00307-1

[39] Brown S and Tuffery R (2010) Induction of alternative oxidase activity in *Candida albicans* by oxidising conditions. Int J Biol Life Sci 6, 26–30.

[40] Vanlerberghe GC , Cvetkovska M and Wang J (2009) Is the maintenance of homeostatic mitochondrial signaling during stress a physiological role for alternative oxidase? Physiol Plant 137, 392–406. 19549065 10.1111/j.1399-3054.2009.01254.x

[41] Zhang DW , Xu F , Zhang ZW , Chen YE , Du JB , Jia SD , Yuan S and Lin HH (2010) Effects of light on cyanide‐resistant respiration and alternative oxidase function in *Arabidopsis* seedlings. Plant Cell Environ 33, 2121–2131. 20716069 10.1111/j.1365-3040.2010.02211.x

[42] Zhang DW , Yuan S , Xu F , Zhu F , Yuan M , Ye HX , Guo HQ , Lv X , Yin Y and Lin HH (2016) Light intensity affects chlorophyll synthesis during greening process by metabolite signal from mitochondrial alternative oxidase in *Arabidopsis* . Plant Cell Environ 39, 12–25. 25158995 10.1111/pce.12438

[43] Saisho D , Nambara E , Naito S , Tsutsumi N , Hirai A and Nakazono M (1997) Characterization of the gene family for alternative oxidase from *Arabidopsis thaliana* . Plant Mol Biol 35, 585–596. 9349280 10.1023/a:1005818507743

[44] Saisho D , Nakazono M , Tsutsumi N and Hirai A (2001) ATP synthesis inhibitors as well as respiratory inhibitors increase steady‐state level of alternative oxidase mRNA in *Arabidopsis thaliana* . J Plant Physiol 158, 241–245.

[45] Clifton R , Millar AH and Whelan J (2006) Alternative oxidases in *Arabidopsis*: a comparative analysis of differential expression in the gene family provides new insights into function of non‐phosphorylating bypasses. Biochim Biophys Acta 1757, 730–741. 16859634 10.1016/j.bbabio.2006.03.009

[46] Swathi M , Lokya V , Swaroop V , Mallikarjuna N , Kannan M , Dutta‐Gupta A and Padmasree K (2014) Structural and functional characterization of proteinase inhibitors from seeds of *Cajanus cajan* (cv. ICP 7118). Plant Physiol Biochem 83, 77–87. 25093261 10.1016/j.plaphy.2014.07.009

[47] Laskar S , Bhattacharyya MK , Shankar R and Bhattacharyya S (2011) *HSP90* controls *SIR2* mediated gene silencing. PLoS One 6, e23406. 21829731 10.1371/journal.pone.0023406 PMC3150437

[48] Luhua S , Ciftci‐Yilmaz S , Harper J , Cushman J and Mittler R (2008) Enhanced tolerance to oxidative stress in transgenic *Arabidopsis* plants expressing proteins of unknown function. Plant Physiol 148, 280–292. 18614705 10.1104/pp.108.124875 PMC2528079

[49] Agrimi G , Brambilla L , Frascotti G , Pisano I , Porro D , Vai M and Palmieri L (2011) Deletion or overexpression of mitochondrial NAD^+^ carriers in *Saccharomyces cerevisiae* alters cellular NAD and ATP contents and affects mitochondrial metabolism and the rate of glycolysis. Appl Environ Microbiol 77, 2239–2246. 21335394 10.1128/AEM.01703-10 PMC3067453

[50] Dalal A , Vishwakarma A , Singh NK , Gudla T , Bhattacharyya MK , Padmasree K , Viehhauser A , Dietz KJ and Kirti PB (2014) Attenuation of hydrogen peroxide‐mediated oxidative stress by *Brassica juncea* annexin‐3 counteracts thiol‐specific antioxidant (TSA1) deficiency in *Saccharomyces cerevisiae* . FEBS Lett 588, 584–593. 24444602 10.1016/j.febslet.2014.01.006

[51] Jang HH , Lee KO , Chi YH , Jung BG , Park SK , Park JH , Lee JR , Lee SS , Moon JC , Yun JW *et al* (2004) Two enzymes in one; two yeast peroxiredoxins display oxidative stress‐dependent switching from a peroxidase to a molecular chaperone function. Cell 117, 625–635. 15163410 10.1016/j.cell.2004.05.002

[52] Queval G and Noctor G (2007) A plate reader method for the measurement of NAD, NADP, glutathione, and ascorbate in tissue extracts: Application to redox profiling during *Arabidopsis* rosette development. Anal Biochem 363, 58–69. 17288982 10.1016/j.ab.2007.01.005

[53] Schmitt ME , Brown TA and Trumpower BL (1990) A rapid and simple method for preparation of RNA from *Saccharomyces cerevisiae* . Nucleic Acids Res 18, 3091–3092. 2190191 10.1093/nar/18.10.3091 PMC330876

[54] Livak KJ and Schmittgen TD (2001) Analysis of relative gene expression data using real‐time quantitative PCR and the 2^‐ΔΔCT^ method. Methods 25, 402–408. 11846609 10.1006/meth.2001.1262

[55] Mittler R (2002) Oxidative stress, antioxidants and stress tolerance. Trends Plant Sci 7, 405–410. 12234732 10.1016/s1360-1385(02)02312-9

[56] Amirsadeghi S , Robson CA , McDonald AE and Vanlerberghe GC (2006) Changes in plant mitochondrial electron transport alter cellular levels of reactive oxygen species and susceptibility to cell death signaling molecules. Plant Cell Physiol 47, 1509–1519. 17012741 10.1093/pcp/pcl016

[57] Igamberdiev AU , Ratcliffe RG and Gupta KJ (2014) Plant mitochondria: source and target for nitric oxide. Mitochondrion 19, 329–333. 24561220 10.1016/j.mito.2014.02.003

[58] Sieger SM , Kristensen BK , Robson CA , Amirsadeghi S , Eng EW , Abdel‐Mesih A , Møller IM and Vanlerberghe GC (2005) The role of alternative oxidase in modulating carbon use efficiency and growth during macronutrient stress in tobacco cells. J Exp Bot 56, 1499–1515. 15824074 10.1093/jxb/eri146

[59] Padmasree K and Raghavendra AS (1999) Importance of oxidative electron transport over oxidative phosphorylation in optimizing photosynthesis in mesophyll protoplasts of pea (*Pisum sativum* L.). Physiol Plant 105, 546–553.

[60] Dinakar C , Abhaypratap V , Yearla SR , Raghavendra AS and Padmasree K (2010) Importance of ROS and antioxidant system during the beneficial interactions of mitochondrial metabolism with photosynthetic carbon assimilation. Planta 231, 461–474. 19943171 10.1007/s00425-009-1067-3

[61] Vanlerberghe GC (2013) Alternative oxidase: a mitochondrial respiratory pathway to maintain metabolic and signaling homeostasis during abiotic and biotic stress in plants. Int J Mol Sci 14, 6805–6847. 23531539 10.3390/ijms14046805 PMC3645666

[62] Matsukawa K , Kamata T and Ito K (2009) Functional expression of plant alternative oxidase decreases antimycin A‐induced reactive oxygen species production in human cells. FEBS Lett 583, 148–152. 19059403 10.1016/j.febslet.2008.11.040

[63] Rogov A and Zvyagilskaya R (2015) Physiological role of alternative oxidase (from yeasts to plants). Biochemistry (Moscow) 80, 400–407. 25869356 10.1134/S0006297915040021

[64] Minagawa N and Yoshimoto A (1987) The induction of cyanide‐resistant respiration in *Hansenula anomala* . J Biochem 101, 1141–1146. 3654588 10.1093/oxfordjournals.jbchem.a121978

[65] Vanlerberghe GC , Robson CA and Yip JY (2002) Induction of mitochondrial alternative oxidase in response to a cell signal pathway down‐regulating the cytochrome pathway prevents programmed cell death. Plant Physiol 129, 1829–1842. 12177496 10.1104/pp.002691 PMC166771

[66] Barros MH , Bandy B , Tahara EB and Kowaltowski AJ (2004) Higher respiratory activity decreases mitochondrial reactive oxygen release and increases life span in *Saccharomyces cerevisiae* . J Biol Chem 279, 49883–49888. 15383542 10.1074/jbc.M408918200

[67] Finkel T (2003) Oxidant signals and oxidative stress. Curr Opin Cell Biol 15, 247–254. 12648682 10.1016/s0955-0674(03)00002-4

[68] Apel K and Hirt H (2004) Reactive oxygen species: metabolism, oxidative stress, and signal transduction. Annu Rev Plant Biol 55, 373–399. 15377225 10.1146/annurev.arplant.55.031903.141701

[69] Asada K (2006) Production and scavenging of reactive oxygen species in chloroplasts and their functions. Plant Physiol 141, 391–396. 16760493 10.1104/pp.106.082040 PMC1475469

[70] Perrone GG , Tan S‐X and Dawes IW (2008) Reactive oxygen species and yeast apoptosis. Biochim Biophys Acta (BBA)‐Mol. Cell Res 1783, 1354–1368. 10.1016/j.bbamcr.2008.01.023 18298957

[71] Farrugia G and Balzan R (2012) Oxidative stress and programmed cell death in yeast. Front Oncol 2, 64. 22737670 10.3389/fonc.2012.00064 PMC3380282

[72] Ayer A , Gourlay CW and Dawes IW (2014) Cellular redox homeostasis, reactive oxygen species and replicative ageing in *Saccharomyces cerevisiae* . FEMS Yeast Res 14, 60–72. 24164795 10.1111/1567-1364.12114

[73] Wheeler GL and Grant CM (2004) Regulation of redox homeostasis in the yeast *Saccharomyces cerevisiae* . Physiol Plant 120, 12–20. 15032872 10.1111/j.0031-9317.2004.0193.x

[74] Lin S‐J , Ford E , Haigis M , Liszt G and Guarente L (2004) Calorie restriction extends yeast life span by lowering the level of NADH. Genes Dev 18, 12–16. 14724176 10.1101/gad.1164804 PMC314267

[75] Lin S‐J , Kaeberlein M , Andalis AA , Sturtz LA , Defossez P‐A , Culotta VC , Fink GR and Guarente L (2002) Calorie restriction extends *Saccharomyces cerevisiae* lifespan by increasing respiration. Nature 418, 344–348. 12124627 10.1038/nature00829

[76] Rietsch A and Beckwith J (1998) The genetics of disulfide bond metabolism. Annu Rev Genet 32, 163–184. 9928478 10.1146/annurev.genet.32.1.163

[77] Herrero E , Ros J , Bellí G and Cabiscol E (2008) Redox control and oxidative stress in yeast cells. Biochim Biophys Acta 1780, 1217–1235. 18178164 10.1016/j.bbagen.2007.12.004

[78] Purvis AC (1997) Role of the alternative oxidase in limiting superoxide production by plant mitochondria. Physiol Plant 100, 165–170.

[79] Gelhaye E , Rouhier N , Gérard J , Jolivet Y , Gualberto J , Navrot N , Ohlsson P‐I , Wingsle G , Hirasawa M , Knaff DB *et al* (2004) A specific form of thioredoxin h occurs in plant mitochondria and regulates the alternative oxidase. Proc Natl Acad Sci USA 101, 14545–14550. 15385674 10.1073/pnas.0405282101 PMC521959

[80] Arnholdt‐Schmitt B , Costa JH and de Melo DF (2006) AOX – a functional marker for efficient cell reprogramming under stress? Trends Plant Sci 11, 281–287. 16713324 10.1016/j.tplants.2006.05.001

