# Supplementary material for: Genetic engineering of AtAOX1a in Saccharomyces cerevisiae prevents oxidative damage and maintains redox homeostasis
Source: FEBS Open Bio. 2016 Feb 1;6(2):135–46. doi: 10.1002/2211-5463.12028 (PMC4821348; doi:10.1002/2211-5463.12028)
Supplement: Supplementary file 1 — Fig. S1 MALDI‐TOF‐TOF mass spectrum of trypsin digested purified AtAOX1a protein between 500 and 5000 m/z. Fig. S2 Lift spectrum and Biotools display of four major peaks from trypsin digested AtAOX1a protein: (A) m/z 1209.819, (B) 1659.057, (C) 1899.286, and (D) 2384.592. [file FEB4-6-135-s001.docx]

**Supplemental Figure**

**Fig.1** MALDI-TOF-TOF mass spectrum of trypsin digested purified AtAOX1a protein between 500-5,000 m/z. The prominent peaks corresponding to 1209.819, 1659.057, 1899.286 and 2384.592 Da were subjected to MS-MS analysis.

**Fig.2** Lift spectrum and Biotools display of four major peaks from trypsin digested AtAOX1a protein: (A) m/z 1209.819, (B) 1659.057, (C) 1899.286 and (D) 2384.592. The sequence obtained from these peptides showed 100% matching with internal sequences of Arabidopsis AOX1a (AT3G22370) as described in Figure 1B.

**Supplemental Figure**

**Fig. S1**


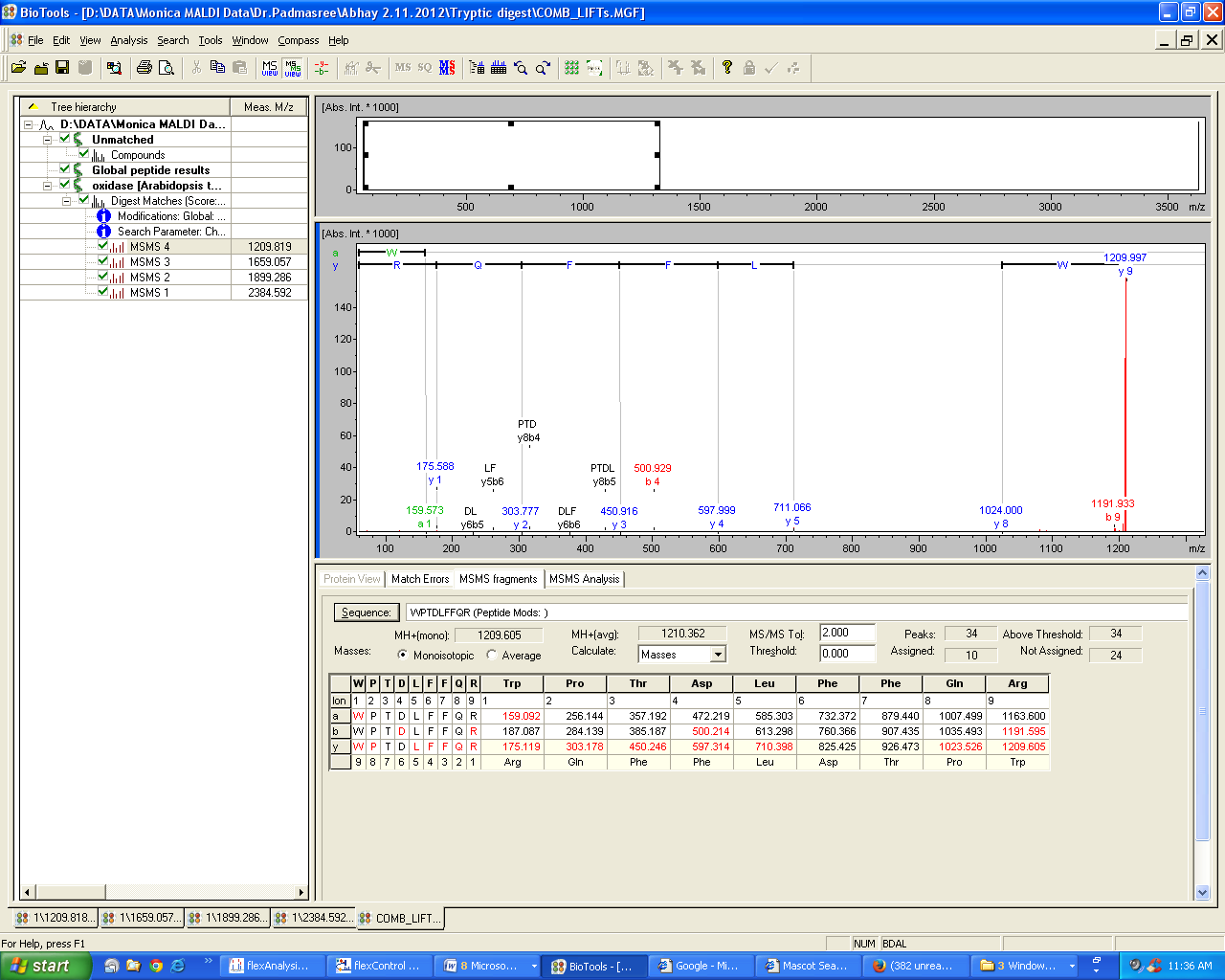


**A**


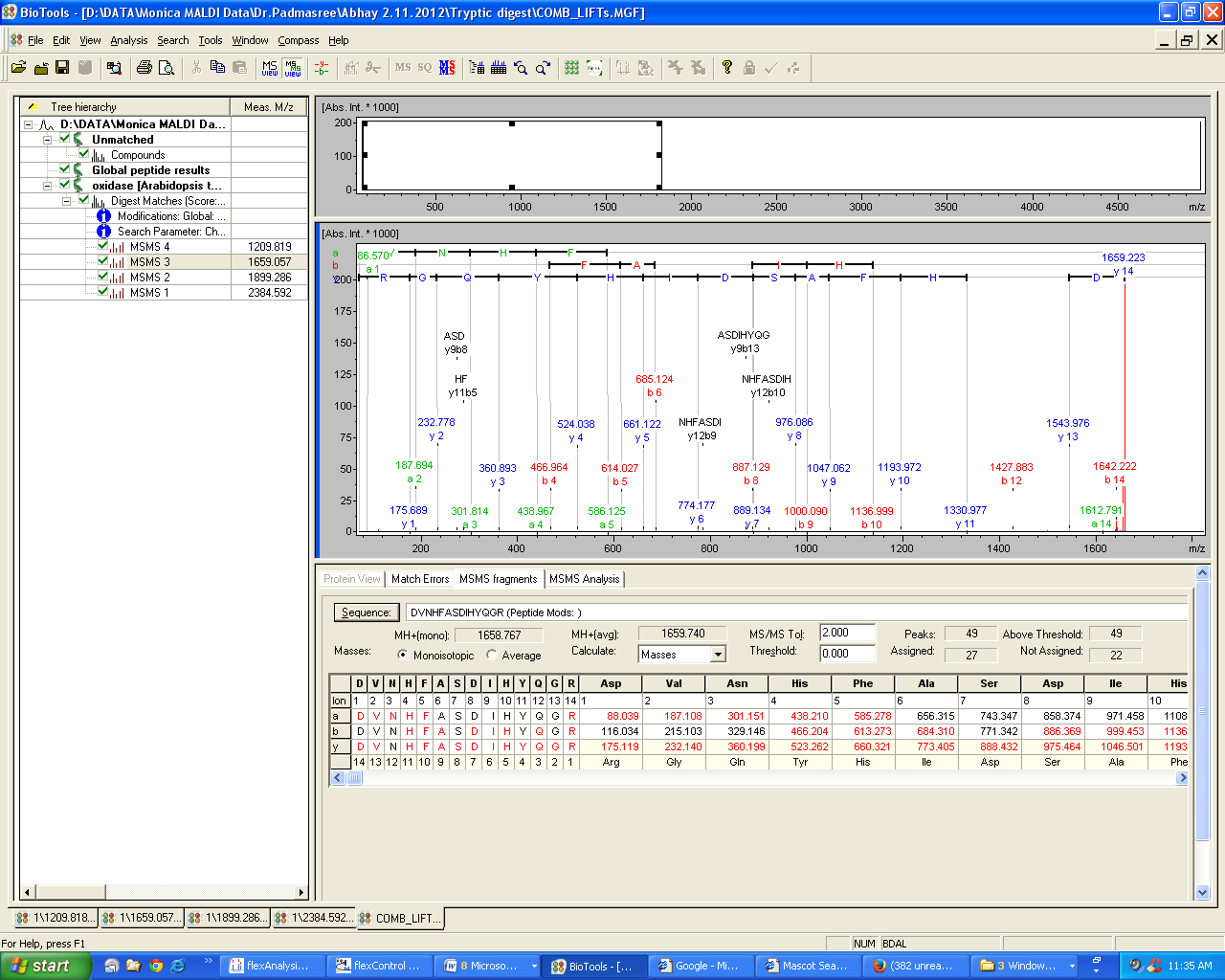


**B**

**Fig. S2**


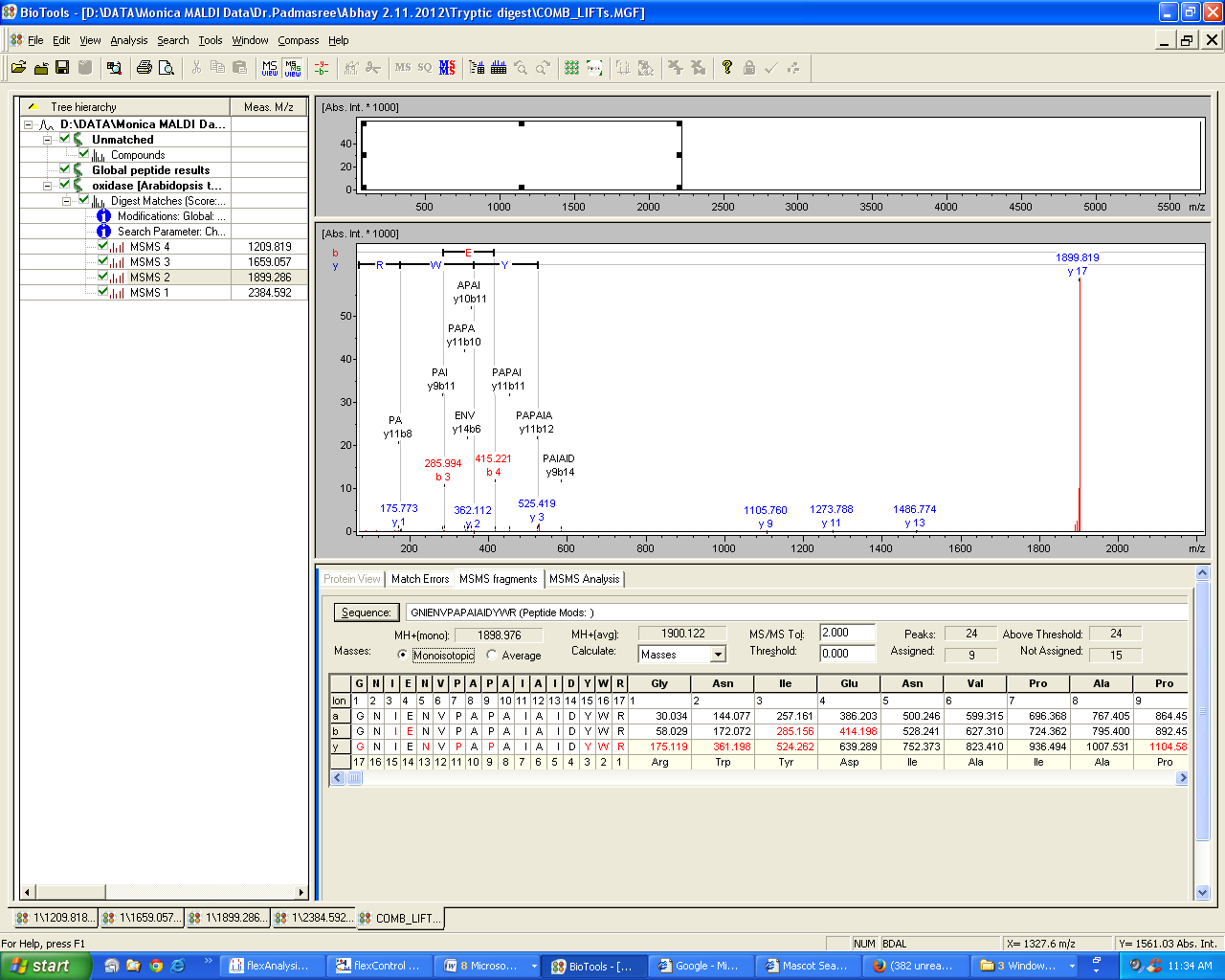


**C**


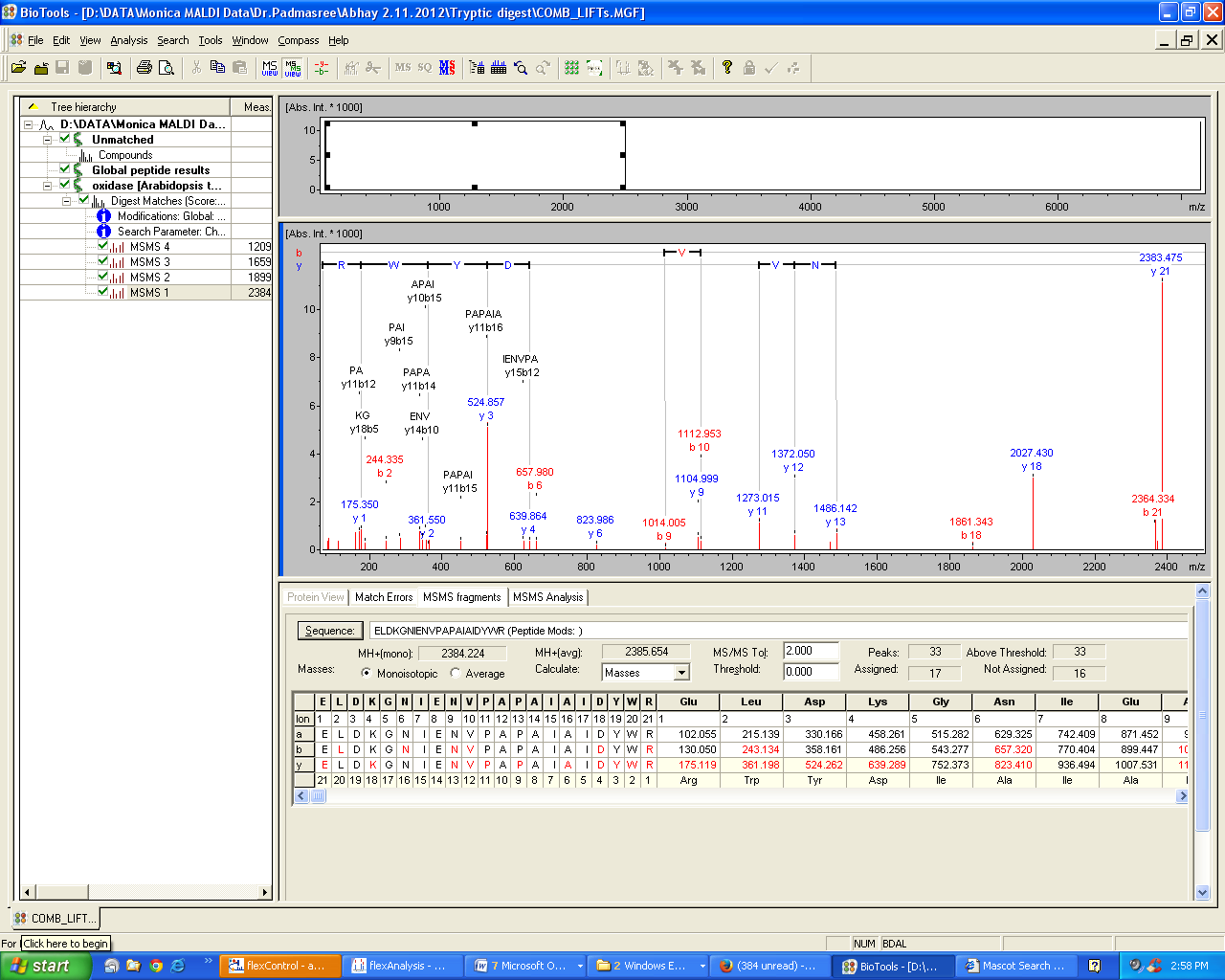


**D**

**Fig. S2**
